# Identifying regulators of parental imprinting by CRISPR/Cas9 screening in haploid human embryonic stem cells

**DOI:** 10.1038/s41467-021-26949-7

**Published:** 2021-11-18

**Authors:** Shiran Bar, Dan Vershkov, Gal Keshet, Elyad Lezmi, Naama Meller, Atilgan Yilmaz, Ofra Yanuka, Malka Nissim-Rafinia, Eran Meshorer, Talia Eldar-Geva, Nissim Benvenisty

**Affiliations:** 1 The Azrieli Center for Stem Cells and Genetic Research, Department of Genetics, The Alexander Silberman Institute of Life Sciences, The Hebrew University of Jerusalem, Edmond J. Safra Campus, Givat Ram, Jerusalem, 91904 Israel; 2 Department of Genetics, The Alexander Silberman Institute of Life Sciences, The Hebrew University of Jerusalem, Edmond J. Safra Campus, Givat Ram, Jerusalem, 91904 Israel; 3 Edmond and Lily Center for Brain Sciences (ELSC), The Hebrew University of Jerusalem, Jerusalem, 91904 Israel; 4 IVF Unit, Division of Obstetrics and Gynecology, Shaare Zedek Medical Center, Jerusalem, 91031 Israel; 5 The Hebrew University School of Medicine, Jerusalem, 91120 Israel

**Keywords:** Imprinting, Embryonic stem cells, Imprinting, DNA methylation

## Abstract

In mammals, imprinted genes are regulated by differentially methylated regions (DMRs) that are inherited from germ cells, leading to monoallelic expression in accordance with parent-of-origin. Yet, it is largely unknown how imprinted DMRs are maintained in human embryos despite global DNA demethylation following fertilization. Here, we explored the mechanisms involved in imprinting regulation by employing human parthenogenetic embryonic stem cells (hpESCs), which lack paternal alleles. We show that although global loss of DNA methylation in hpESCs affects most imprinted DMRs, many paternally-expressed genes (PEGs) remain repressed. To search for factors regulating PEGs, we performed a genome-wide CRISPR/Cas9 screen in haploid hpESCs. This revealed *ATF7IP* as an essential repressor of a set of PEGs, which we further show is also required for silencing sperm-specific genes. Our study reinforces an important role for histone modifications in regulating imprinted genes and suggests a link between parental imprinting and germ cell identity.

## Abstract (web summary)

Genetic imprinting ensures monoallelic gene expression critical for normal embryonic development. Here the authors take advantage of human haploid parthenogenic embryonic stem cells lacking paternal alleles to identify, by genome-wide screening, factors involved in the regulation of imprinted genes.

## Introduction

Parental imprinting is a unique epigenetic phenomenon in mammals, involving a group of genes that are only expressed from one parental allele, while the other allele is silenced. Proper expression of imprinted genes is essential for mammalian development, as uniparental embryos having a maternal-only (parthenogenetic) or paternal-only (androgenetic) genomes die during gestation^1,2^. This notion established imprinting as being the block for asexual reproduction in mammals. Moreover, inappropriate expression of imprinted genes leads to various developmental disorders^3,4^ and is also involved in cancer progression^5^.

The monoallelic expression of imprinted genes is regulated by differentially methylated regions (DMRs) that are established in the germline. Shortly after fertilization, the mammalian genome is extensively demethylated and by the time it reaches the blastocyst stage, most parental DNA methylation patterns are erased. Nevertheless, imprinted germline DMRs are exceptionally maintained during this time, preserving allelic parental identity^6^. During early embryogenesis, “secondary” DMRs may appear at imprinted genes, while other “transient” DMRs disappear within days after fertilization^3^. Yet, the factors regulating the maintenance of imprinting during human embryogenesis are mostly unknown^7^.

Multi-locus imprinting disturbances (MLID) result in DNA hypomethylation of several imprinted loci (HIL). This phenomenon was identified in patients diagnosed with imprinting diseases which are caused by aberrant DNA methylation, such as Beckwith-Wiedemann and Russel-Silver syndromes^8–12^. It is hypothesized that MLID patients carry mutations in genes involved in imprinting regulation, however for most cases, the identity of such factors has yet to be identified^13^. MLID occurs in some patients diagnosed with Transient-Neonatal Diabetes Mellitus (TNDM) and is driven by homozygous mutations in the zinc finger protein gene ZFP57. These patients exhibit methylation aberrations in several DMRs (e.g., PLAGL1 and GRB10)^14^, yet other TNDM patients who do not carry ZFP57 mutations experience hypomethylation in many additional DMRs^15,16^, which is driven by an unknown mechanism. In mouse embryonic stem cells (ESCs), ZFP57 was shown to regulate multiple imprinted loci^17–21^ and recently, the zinc finger protein ZNF445 was also suggested to be involved in imprinting regulation^22^. Nevertheless, while extensive loss-of-imprinting occurs in mice following knockout of both Zfp57 and Znf445, most imprinted DMRs are still preserved in human ESCs following knockdown of these genes^22^. In addition, PGC7/STELLA^21,23,24^ and G9a/GLP^25^ were shown to be important for the preservation of several DMRs in mouse ESCs. Nevertheless, their effect on imprinting in humans has not been thoroughly examined. Collectively, accumulating evidence suggests that imprinting might be regulated by multiple factors in a species-specific manner^7^.

Here, we use haploid and diploid parthenogenetic human ESCs (hpESCs)^26^ to perform a comprehensive analysis of imprinting regulation. We employed a CRISPR/Cas9 loss-of-function screening to identify factors involved in this process. hpESCs serve as a favorable tool to study imprinting in humans for several reasons: (1) hpESCs consist of only the maternal genome. Thus, imprinted paternally expressed genes (PEGs) are silenced in these cells, markedly simplifying interrogation of imprinting. (2) hESCs are widely used to model human embryonic development because of their pluripotent nature^27^. (3) Using haploid cells enhances the efficiency of loss-of-function screens^26,28^. Exploiting these advantages, we investigated both known and unknown mechanisms that are required for maintaining parental imprinting.

## Results

### Global DNA demethylation in hpESCs activates only a subset of imprinted genes

DNA methylation is considered a central mechanism for the monoallelic silencing of imprinted genes^29^. To explore this mode of imprinting regulation, we analyzed the consequences of global DNA demethylation achieved by both genetic and pharmacological manipulations. To this end, we applied CRISPR/Cas9 to genetically target the DNA methyltransferase 1 (DNMT1) enzyme and used the DNMT inhibitor 5-aza-2′-deoxycytidine (5-azadC) to chemically induce hypomethylation in hpESCs. While mouse ESCs were shown to retain proper self-renewal upon a triple knockout (KO) of *Dnmt1*/*Dnmt3a*/*Dnmt3b*^30^, human ESCs are sensitive to the loss of DNMT1^31^. Indeed, several days after the initiation of demethylation (by infection with lentivirus encoding sgRNA targeting *DNMT1*, or following treatment with 5-azadC), there was an apparent cell death in the hpESC culture. However, we were able to optimize the experimental conditions to yield efficient infection and quick selection, which allowed the collection of viable cells for analysis of gene expression and CpG methylation profiles.

**Fig. 1:**
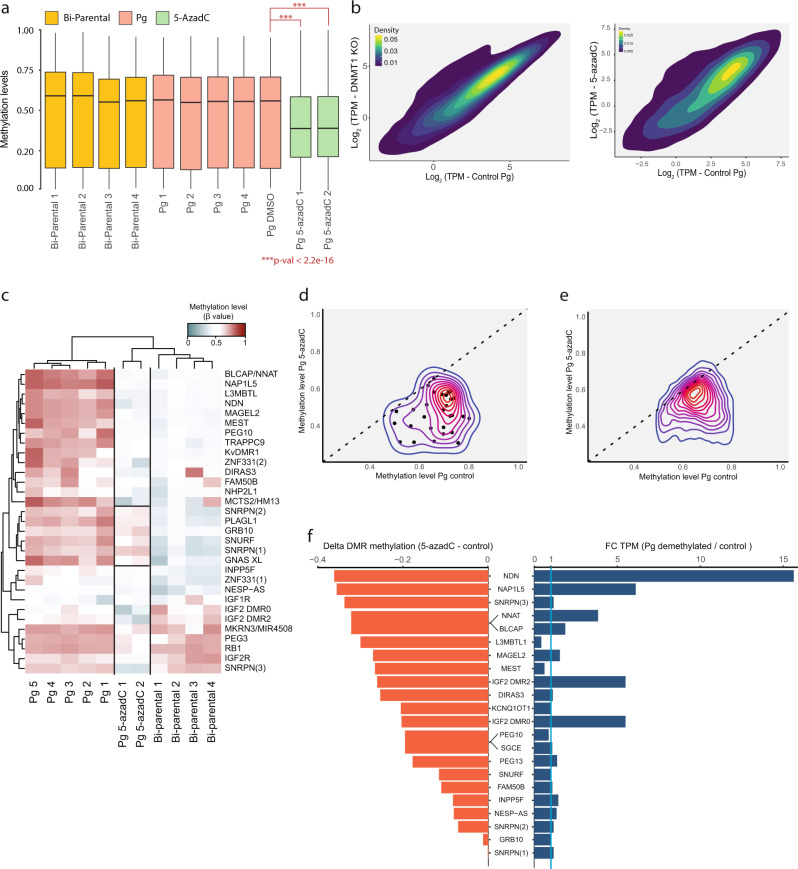
Global DNA demethylation in parthenogenetic human ESCs. **a** Boxplots displaying the median with 25th–75th percentile and range of global DNA methylation levels (β values) across all analyzed CpGs in bi-parental hESCs (yellow), control parthenogenetic hESCs (red) and 5-azadC-treated parthenogenetic hESCs (green). ^***^P < 2.2e-16 (one-tailed, paired *t*-test). Whiskers represent values within 1.5 X the interquartile range. **b** 2D density plot showing the log_2_ TPM of all genes in DNMT1-KO (left *y* axis) or 5-azadC-treated hpESCs (right *y* axis) vs. control hpESCs (empty Cas9 vector or DMSO, *x* axis). **c** Heatmap of mean methylation levels (β values) of maternal DMRs in bi-parental, control parthenogenetic and 5-azadC-treated hpESCs. **d** 2D density plot showing mean methylation levels (β values) of regions associated with maternal DMRs in control (*x* axis) vs. 5-azadC-treated (*y* axis) hpESCs. Only regions with mean methylation >0.5 in control hpESCs are included. Dashed line represents identical methylation (slope = 1). **e** Same analysis as in (**c**), performed for genome-wide regions associated with transcription start sites (TSS), 5′-UTR and 1st exon. **f** Two-sided bar plot of PEGs that are expressed in bi-parental hESCs. Left bars (orange) illustrate the delta methylation levels (β values) of the associated maternal DMR, between 5-azadC-treated and control hpESCs. Wide bars are used for DMRs that control several PEGs. Right bars (blue) represent fold change (FC) of the mean TPM between DNMT1 KO and 5-azadC-treated (referred to as demethylated) vs. control hpESCs. Blue line illustrates unchanged expression (FC = 1).

5-azadC treatment resulted in a significant reduction of global DNA methylation levels (Fig. 1a, *P* < 2.2e-16, one-tailed, paired *t*-test) and an upregulation of a subset of genes (Fig. 1b). *DNMT1* KO also induced a notable overexpression of a group of genes, similar to those that were affected by 5-azadC (Fig. 1b and Supplementary Fig. 1a). Combining *DNMT1* KO and 5-azadC-treated samples for a differential expression analysis of hypomethylated vs. control hpESCs revealed significantly upregulated and downregulated genes following demethylation, which were mostly associated with gene ontology (GO) terms related to methylation silencing and KRAS activation (Supplementary Fig. 1b, c).

While paternal DMRs are usually found in intergenic regions, maternal DMRs are commonly based at gene promoters and are thought to mainly regulate imprinting in *cis*. Following 5-azadC treatment, most maternal DMRs (which are hypermethylated in parthenogenetic cells) were extensively demethylated (Fig. 1 c, d), similar to the genome-wide effect observed for hypermethylated promoters (Fig. 1e). Nevertheless, several DMRs (e.g., *SNRPN* DMRs 1 & 2, *GRB10*, *PLAGL1*, *GNAS-XL*) retained relatively high DNA methylation levels (Fig. 1c), suggesting differential sensitivity to global demethylation among maternal DMRs. The evident loss of methylation in the remaining maternal DMRs (Fig. 1c), induced a subsequent expression in some PEGs, but most of them remained silenced (58%, 11/19 PEGs; Fig. 1f and Supplementary Fig. 1d, e). Some PEGs that were activated following demethylation (e.g., NDN, NNAT) were transcribed at similar levels as in bi-parental cells, whereas the expression of others (e.g., NAP1L5) were still much lower than in bi-parental cells (Supplementary Fig. 1f). Together, these results reveal distinct responses to DNMT1 inhibition among PEGs, both in the extent of demethylation, as well as in the transcriptional consequences of hypomethylation. This suggests that the mode by which DNA methylation regulates imprinting in humans is locus specific. Notably, the repressive state of many PEGs is retained, even when their associated DMRs were hypomethylated. This implies the existence of additional regulatory layers which might repress the expression of these PEGs on the maternal allele.

### Genome-wide CRISPR/Cas9 screen to identify factors that maintain imprinting of PEG10

**Fig. 2:**
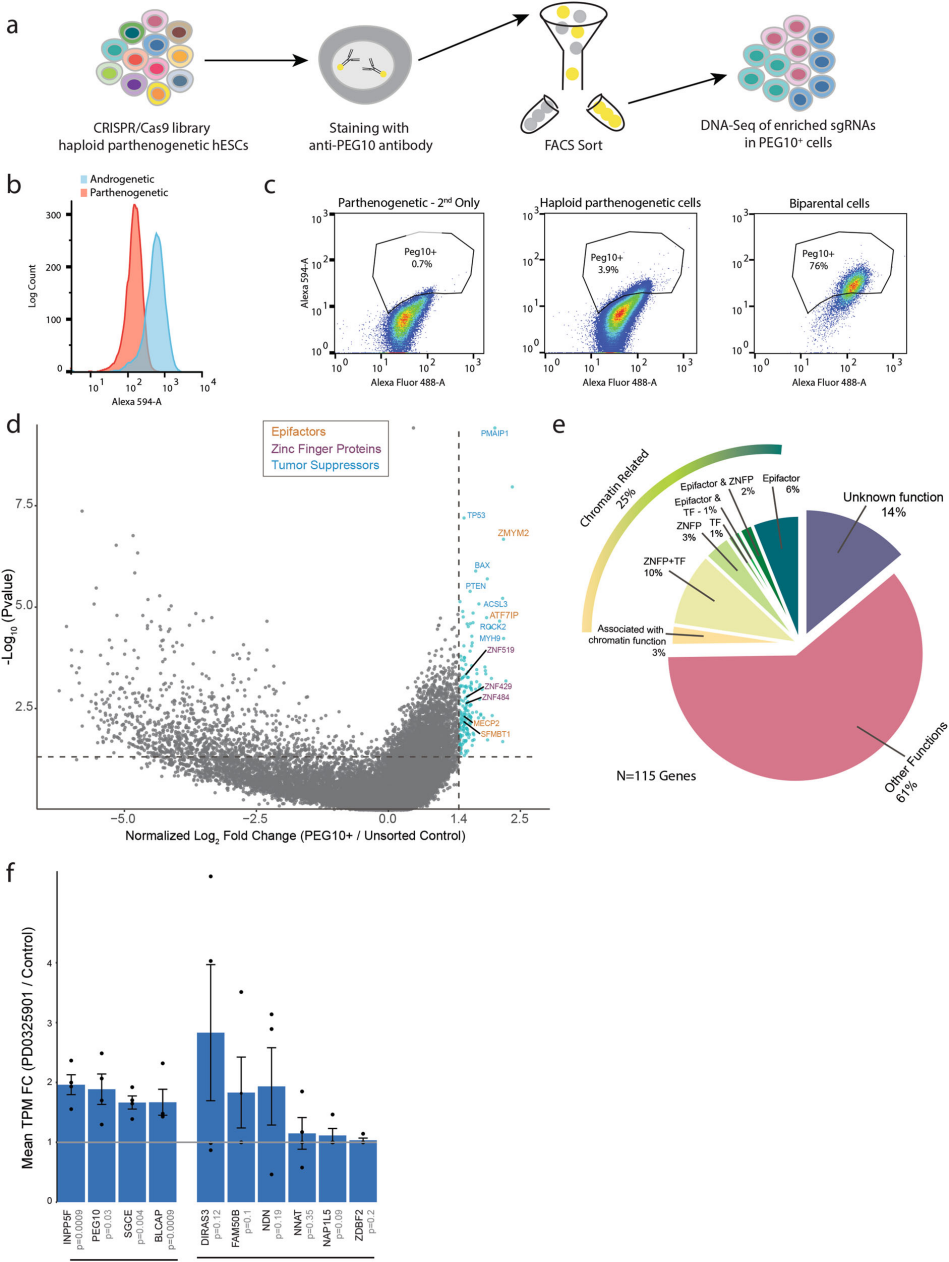
Loss-of-function screen in haploid hESCs reveals regulators of imprinting. **a** Schematic overview of the loss-of-function screen experimental setup. Haploid parthenogenetic hESCs infected with a genome-wide CRISPR/Cas9 sgRNA library, were stained with an antibody against the paternally expressed gene PEG10 (normally silenced in these cells). This was followed by FACS sorting to isolate the PEG10-positive (PEG10^+^) cell population. Finally, targeted DNA sequencing of sgRNAs identifies enriched genes mutated in PEG10^+^ cells compared with unsorted control. **b** Histogram showing the flow cytometry analysis of PEG10 staining in androgenetic (blue) vs. parthenogenetic (red) cells. **c** Representative scatter plots of the flow cytometry analysis of PEG10 staining in secondary only control (left), haploid parthenogenetic CRISPR/Cas9 library cells (center) and bi-parental cells (right). *y* Axis represents the fluorescence intensity of PEG10 staining, while the *x* axis represents the autofluorescence signal. **d** Volcano plot showing the median log_2_ fold change (FC) of normalized sgRNA read counts (calculated by edgeR) per gene, between PEG10^+^ and unsorted control (*x* axis, values are normalized to zero. *n* = 4 replicate screens). *y* Axis represents −log_10_ of the *P* value (two-sample, two-sided Kolmogorov-Smirnov test). Marked in blue are enriched genes having log FC > 0.5 (equivalent to normalized value >1.4) and *P* value < 0.05. Representative genes included in the “EpiFactors” database (orange), zinc finger proteins (purple) and tumor suppressors (blue) are indicated. **e** Pie chart subgrouping the 115 candidate genes by function (after removing genes enriched in the PEG10-negative control). Chromatin-related genes are further divided to subcategories: Genes included in the EpiFactors database, genes encoding zinc finger proteins (ZNFP) and those encoding transcription factors (TF). **f** Mean expression FC of PEGs between hpESCs treated with the MEK/ERK inhibitor PD0325901 and DMSO. *n* = 4 replicates from each treatment in two different cell lines. Data are presented as mean ± SEM. Shown are PEGs with FC > 1. *P* values are listed in gray (one-tailed, paired *t*-test).

To search for unknown factors that maintain imprinting, we conducted a CRISPR/Cas9 genome-wide loss-of-function screen in haploid hpESCs (Fig. 2a). We focused on the imprinted gene *PEG10*, because it is a single-isoform imprinted gene which is silenced in hpESCs^32^ and is not significantly activated following demethylation (Fig. 1e, f). Moreover, *PEG10* is important for normal placental development^33^ and is overexpressed in various cancers, promoting tumor proliferation^34^. To isolate cells that activate *PEG10*, we performed intracellular immunofluorescent staining using anti-PEG10 antibody. FACS analysis of immunostained samples was able to distinguish between androgenetic cells (expressing *PEG10*) and parthenogenetic cells (in which *PEG10* is silenced) (Fig. 2b). Next, we applied this immunostaining to the CRISPR/Cas9 parthenogenetic library that consist ~180,000 sgRNAs targeting 18,166 genes^28^ (~150 × 10^6^ cells were stained in each replicate, >800 fold of the library size). Immunostained library cells were sorted using FACS to collect the small PEG10-positive (PEG10^+^) population (~4 × 10^5^−1 × 10^6^ cells), as well as a comparable fraction of PEG10-negative cells. The distribution of sgRNAs within the sorted samples were assessed using high-throughput sequencing and compared to the unsorted library to identify candidate genes whose perturbations resulted in *PEG10* activation (Fig. 2a, c, d). We removed genes that were significantly enriched also in the PEG10-negative sorted samples, thereby eliminating multiple tumor suppressors (Supplementary Fig. 2a, b, see “Methods” for more details). Finally, we established a list of 115 significantly enriched genes that are potentially important for maintaining maternal imprinting at the *PEG10* locus (referred to as “candidate genes”; Supplementary Data File 1). These candidate genes were enriched in GO terms associated mainly with transcription, DNA binding, and repressive functions (Supplementary Fig. 2a) and a subset of them were functionally related to chromatin, appearing in the EpiFactors database^35^, identified as zinc finger proteins (ZNFP, taken from www.genenames.org), transcription factors (TF)^36^, or other chromatin-related roles (Fig. 2e). Genes listed in the EpiFactors database were specifically enriched in the candidate list compared to their representation in the entire library (*P* = 0.027, Fisher’s exact test). The identification of many chromatin regulators within the candidate genes is consistent with the expected outcomes of the screen to discover factors that repress imprinted genes. Nonetheless, some enriched genes had other functions that are not directly related with chromatin regulation but could still affect imprinting indirectly via various pathways.

### Inhibiting the MEK/ERK pathway in hpESCs drives loss of imprinting

We first searched for upstream global pathways that might be associated with the candidate genes using the Transcription Factor Enrichment Analysis (TFEA) from the X2K Web tool^37^ which labels upstream regulatory networks from a supplied list of genes, followed by Kinase Enrichment Analysis (KEA3)^38^. This analysis identified MAPK1 (ERK2) as the most significant regulator of a transcriptional network involving a set of candidate genes. Another member of the MEK/ERK pathway, MAPK3 (ERK1), was also found among the top 20 enriched kinases (Supplementary Table 1). Moreover, the functions of several candidate genes are directly linked with this pathway (e.g., CAMKV, MFAP3L, FAM83A). Inhibiting MEK/ERK is part of the 2i protocol for establishing naive mESCs^39^, which are characterized by genome-wide DNA hypomethylation^40^ and often exhibit imprinting aberrations^41^. Recent efforts established multiple protocols to generate naive human pluripotent cells, all of which involve inhibition of the MEK/ERK pathway^42^. These cells were also found to exhibit loss-of-imprinting in various regions^43^. To test the involvement of FGF signaling and the MEK/ERK pathway in human imprinting regulation, we treated hpESCs with a specific MEK/ERK inhibitor (PD0325901, 25 μM) for 5 days, in the absence of bFGF. This resulted in significant upregulation of *PEG10*, as suggested by our screen (Fig. 2f). Notably, several additional PEGs were also activated upon MEK/ERK inhibition, namely *INPP5F*, *SGCE*, and *BLCAP* (Fig. 2f).

### ATF7IP regulates PEGs by facilitating the repressive histone modification H3K9me3

**Fig. 3:**
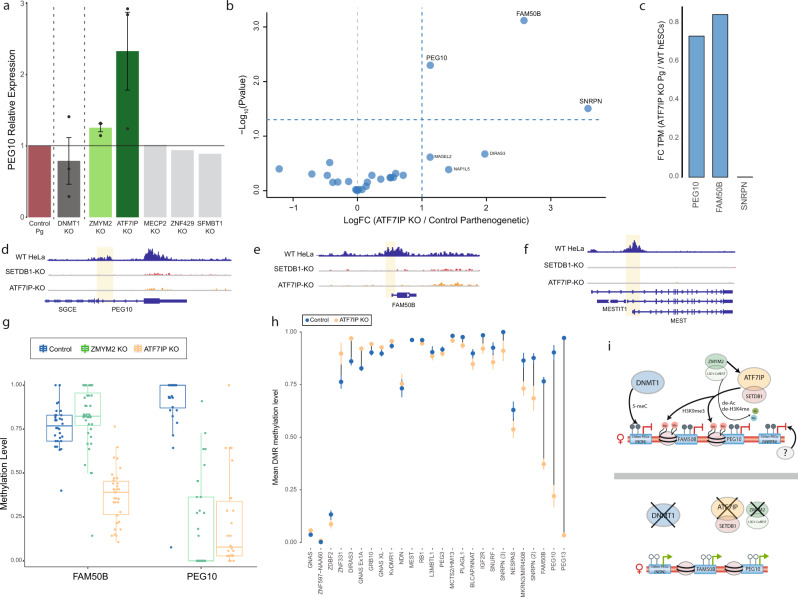
ATF7IP is required to maintain a set of maternally imprinted loci. **a** Bar plot of *PEG10* expression relative to control, in hpESCs cells infected with sgRNAs targeting *DNMT1* and 5 candidate genes. *n* = 3 independent experiments for DNMT1, ATF7IP, ZMYM2, and control samples. Data are presented as mean ± SEM. **b** Volcano plot of differential expression (calculated by edgeR) showing the log_2_ fold change (FC) between *ATF7IP* KO and control (empty Cas9 vector) hpESCs for all PEGs (*x* axis). *n* = 3 replicates from each cell type. *y* Axis represents −log_10_ *P* value. **c** Bar plot showing the FC of the mean TPM in ATF7IP KO hpESCs (*n* = 3) relative to the mean TPM in bi-parental hESCs (*n* = 4). **d**–**f** Integrated Genomics Viewer (IGV) visualization of H3K9me3-ChIP-Seq peaks in WT, SETDB1 KO, and ATF7IP KO HeLa cells at the PEG10 locus (**d**), FAM50B (**e**), and MEST (**f**). The location of the imprinted DMR is highlighted in yellow. **g** Boxplots displaying the median with 25th–75th percentile and range of methylation levels (β values) of individual CpGs within the DMRs of *PEG10* and *FAM50B* in control hpESCs (blue), *ZMYM2* KO (green), or *ATF7IP* KO (orange) hpESCs. Dots represent individual CpGs. Whiskers represent values within 1.5 times the interquartile range. **h** Mean methylation levels (β values) of imprinted DMRs in control (blue) or *ATF7IP* KO (orange) hpESCs. Data are presented as mean ± SEM. Lines are drawn between dots to represent the difference in mean methylation levels. **i** An illustration summarizing our model for imprinting regulation in hESCs. Maternal imprinting in hESCs is maintained by various factors in a locus-specific manner. Several PEGs (e.g., NDN) are directly regulated by DNA methylation and their imprinting depends on *DNMT1* expression. ATF7IP preserves imprinting of *PEG10* and *FAM50B* via the repressive histone modification H3k9me3. *ZMYM2* KO is also sufficient for an apparent loss-of-imprinting of *PEG10*, possibly via its direct interaction with ATF7IP or/and excessive acetylation following its deletion. The repression of other PEGs (e.g., *SNRPN*) in hpESCs is either redundant with, or maintained by factors, which are yet unknown.

Next, we selected for validation five candidate genes that were shown to be involved in transcriptional repression. Using CRISPR/Cas9, we mutated each of these genes in hpESCs individually and showed that knocking out *ATF7IP* and *ZMYM2* led to the activation of *PEG10* (Fig. 3a). ATF7IP was shown to bind the histone methyltransferase SETDB1 and is required for catalyzing the heterochromatin mark H3K9me3 in various genomic regions^44,45^. It also interacts with the methyl CpG binding protein MBD1 to repress transcription^46^ and maintain X chromosome inactivation^47^. ZMYM2 associates with the LSD1/Co-REST/HDAC (LCH) repressive complex, which deacetylates histones and demethylates H3K4me3^48,49^. Interestingly, a direct interaction between ATF7IP and ZMYM2 proteins in mESCs has been recently discovered^50^.

Among the top candidate genes, disruption of *ATF7IP* in hpESCs induced the highest levels of *PEG10* expression (Fig. 3a). Hierarchical clustering analysis based on the expression of all genes revealed similar global expression patterns in *ATF7IP*-KO and control hpESCs (Supplementary Fig. 3a), whereas applying this clustering to PEGs only segregated *ATF7IP*-KO from control hpESCs (Supplementary Fig. 3b). This is different than the pattern observed for *DNMT1*-KO or 5-azadC-treated hpESCs, which clustered separately from control in both the genome-wide and PEG-only analyses (Supplementary Fig. 3a, b). This suggests that while DNA demethylation drives global changes in gene expression, the genome-wide effect of ATF7IP disruption is more restricted, with a major impact on expression of PEGs. Differential expression analysis between *ATF7IP*-KO and control hpESCs identified three significantly upregulated PEGs: *PEG10*, *FAM50B*, and *SNRPN* (Fig. 3b). Yet, while *PEG10* and *FAM50B* are highly expressed in *ATF7IP* KO cells, in comparable levels to those observed in bi-parental cells, the activation of SNRPN reaches ~1% of bi-parental cells (Fig. 3c and Supplementary Fig. 3c). A previous study in HeLa cells demonstrated substantial loss of H3K9me3 in several genomic regions following KO of *ATF7IP* or *SETDB1*^44^. We reanalyzed this ChIP-Seq data and focused on H3K9me3 peaks surrounding imprinted DMRs. In agreement with the expression patterns observed in hpESCs, H3K9me3 peaks at the *PEG10* and *FAM50B* DMRs were lost following *ATF7IP* KO (Fig. 3d, e). However, H3K9me3 levels throughout the Prader-Willi syndrome (PWS) locus encompassing *SNRPN*, were similar between WT and *ATF7IP-*KO or *SETDB1*-KO (Supplementary Fig. 3d). Next, we examined changes in H3K9me3 levels at other maternal DMRs, aiming to point to additional PEGs that might be regulated by ATF7IP. Specifically, the DMRs of *MEST*, *KCNQ1OT1*, *PEG3*, and *GRB10*, also exhibited loss of H3K9me3 peaks in *ATF7IP*-KO HeLa cells (Fig. 3f and Supplementary Fig. 3e–g). However, these genes did not exhibit a higher expression following *ATF7IP* KO in hpESCs, possibly because unlike *PEG10* and *FAM50B*, these genes also consist a non-imprinted isoform^32^, or because they are not normally transcribed in hESCs. Other imprinted loci such as *DIRAS3* did not exhibit a reduction in H3K9me3 levels following *ATF7IP* KO (Supplementary Fig. 3h). We also performed ChIP qPCR of H3K9me3 in hpESCs and confirm its reduction at both PEG10 and FAM50B DMRs (Supplementary Fig. 3i). Next, to probe the effect of ATF7IP on DNA methylation, we performed reduced representation bisulfite sequencing (RRBS) and analyzed methylation patterns in *ATF7IP* KO and control hpESCs. Genome-wide analysis revealed that loss of *ATF7IP* results in a small, but significant, reduction of global DNA methylation levels (Supplementary Fig. 4a, *P* < 2.2e-16, one-tailed *t*-test). Focusing on imprinted regions demonstrated considerable hypomethylation at the DMRs of *PEG10* and *FAM50B* in ATF7IP KO hpESCs and bi-parental hESCs (Fig. 3g and Supplementary Fig. 4b–d). Moreover, significant hypomethylation was also observed at the DMR of *PEG13* (Fig. 3h) which was not activated following loss of *ATF7IP*, possibly because it is mostly not expressed in hESCs.

While loss of ATF7IP induced expression of several imprinted regions, *PEG10* was the only imprinted gene that was significantly upregulated in *ZMYM2* KO hpESCs. In agreement with these transcriptional changes, probing DNA methylation in *ZMYM2* KO hpESCs using RRBS, confirmed a significant reduction at the *PEG10* DMR, but not at the *FAM50B* DMR (Fig. 3g and Supplementary Fig. 4b, c). We showed previously that *ZMYM2* KO in bi-parental hESCs induces global histone H3 hyperacetylation across multiple loci^49^. Analysis of these ChIP-Seq data focusing on imprinted regions demonstrated hyperacetylation within the DMR of *PEG10* in *ZMYM2* KO bi-parental samples (Supplementary Fig. 4e).

Altogether, our analysis on imprinting regulation favors a model in which different factors affect distinct imprinted loci by maintaining various layers of repressive chromatin modifications (Fig. 3i). While the monoallelic silencing of some PEGs (e.g., *NDN*) depends on the presence of DNA methylation and *DNMT1*, suppression of other loci (e.g., *PEG10*, *FAM50B*) relies on histone modifications and their associated chromatin-modifying complexes, namely ZMYM2 and ATF7IP. Still, for some PEGs (e.g., *SNRPN*), the mode of regulation and the proteins required to preserve its imprinting are still obscure and call for further research (Fig. 3i).

### ATF7IP represses genes associated with spermatogenesis

**Fig. 4:**
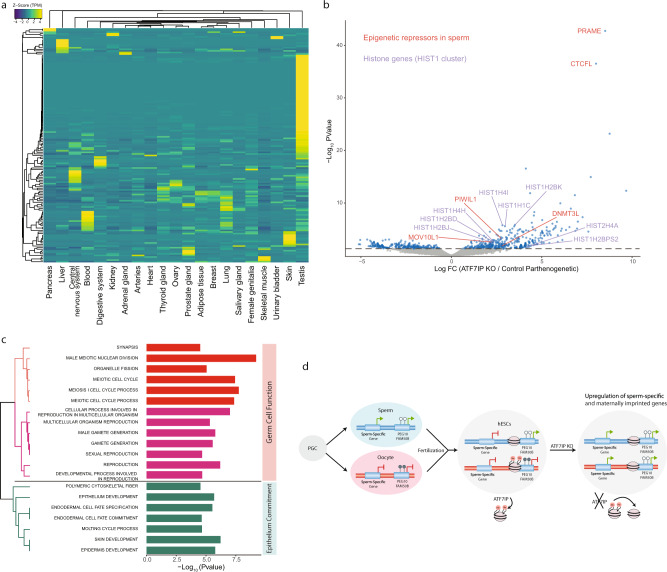
ATF7IP KO induces expression of genes involved in spermatogenesis. **a** Heatmap of expression levels (*Z* score TPM) across tissues (data from the GTEx study) of all the upregulated genes in *ATF7IP* KO cells (158 genes as identified in (**b**), log FC > 2 and *P* value < 0.05). **b** Volcano plot of differential expression (calculated by edgeR) showing the log_2_ fold change (FC) between ATF7IP KO and control (empty Cas9 vector) hpESCs for all genes (*x* axis, *n* = 3 replicates from each cell type). *y* Axis represents −log_10_ *P* value. Marked in blue are genes with *P* value < 0.05. Upregulated genes from the *HIST1* gene cluster are highlighted in purple and representative epigenetic repressors that are mostly active in sperm are shown in red. **c** Top 20 enriched gene ontology (GO) terms (analyzed by GSEA, FDR *q* values < 0.05) among the upregulated protein-coding genes following *ATF7IP* KO (158 genes that were identified in (**b**), log FC > 2 and *P* value < 0.05, excluding *HIST1* genes). *x* Axis represents −log_10_ *P* value (hypergeometric distribution, as calculated by GSEA). Hierarchical clustering for the GO terms was applied based on a matrix of genes associated with each term (“Manhattan” distance measure) and colored accordingly. **d** An illustration summarizing our model for ATF7IP activity in hESCs. Sperm-specific and imprinted paternally expressed genes (PEGs) are differentially expressed between oocyte and sperm and are silenced by ATF7IP in hESCs. PEGs are only repressed from the maternal allele, whereas sperm-specific genes are completely silenced. Following *ATF7IP* KO, these genes are activated, resulting in upregulation of a group of sperm-specific and paternally expressed genes.

Although the broad function of ATF7IP has been studied in cancer cell lines^44,46,51^, its role in hESCs has not been characterized before. To outline the global regulatory activity of ATF7IP in hESCs, we performed a genome-wide differential expression analysis between *ATF7IP*-KO and control hpESCs. This revealed a bias for upregulated genes, consistent with ATF7IP acting mostly as a transcriptional repressor. Analyzing the tissue expression distribution of these upregulated genes using the Genotype-Tissue Expression (GTEx) database revealed a striking large cluster of genes which are specifically expressed in testis (Fig. 4a, b and Supplementary Data File 2). Among these upregulated genes are several epigenetic repressors that are mostly active in sperm (e.g., *PRAME*, *CTCFL*, *DNMT3L*, *PIWIL1*, *MOV10L1*). We also found a significant enrichment of multiple members of the histone gene cluster *HIST1* (*P* < 0.00001, Fisher’s exact test) (Fig. 4b). *HIST1* genes were shown to be highly expressed in normal mitotic spermatogonia in mice^52^ and to be downregulated in mutant spermatogonia cells^53^. Enriched GO terms associated with the upregulated protein-coding genes (discarding *HIST1* genes), identified a marked enrichment for functions related to germ cells, including terms associated with reproduction, male gamete generation and meiosis (Fig. 4c). To ensure that these observations are not restricted to parthenogenetic cells, we directed CRISPR/Cas9 to target *ATF7IP* also in bi-parental hESCs and showed that these cells exhibited similar upregulation of sperm-specific genes (Supplementary Fig. 4a). Additionally, diseases that are associated with ATF7IP mostly involve a testis-related phenotype, and include testicular germ cell tumors, cryptorchidism and male infertility (Supplementary Table 2). Furthermore, inspecting the tissue distribution of the mutations in *ATF7IP* that are reported in the catalogue of somatic mutations in cancer (COSMIC) database demonstrated a significant bias toward copy number variation (CNV) gains in testis. Herein, more than 46% of samples that are associated with testicular cancer exhibited elevated copy numbers of a region on chromosome 12 containing *ATF7IP* (Supplementary Fig. 4b). Overall, our data reveals that loss of ATF7IP in hESCs leads to activation of multiple genes that are specifically expressed in the testis and are associated with spermatogenesis.

## Discussion

Imprinted genes stand out from the rest of the genome, as they are expressed from either the maternal or the paternal allele, depending on DNA methylation inherited from the parental gametes. Although many genes are differentially methylated between oocyte and sperm, most of these regions are reprogrammed during early development, with the exception of imprinted DMRs. Thus, maintenance of DNA methylation during pre-implantation is the decisive step which dictates imprinting. Yet, the mechanisms enabling this maintenance are mostly unknown, especially in humans. Here, we set out to explore these mechanisms, using hpESCs as a model. We describe how global DNA hypomethylation results in distinct consequences on the expression of imprinted genes, and identify regulators of imprinting using a genome-wide loss-of-function screen.

Previous studies have established the essentiality of DNMT1 and DNA methylation maintenance in human ESCs^31^. Accordingly, we observed extensive cell death after targeting DNMT1 with CRISPR/Cas9 or inhibiting it with 5-azadc, but were able to calibrate a short-term experiment to collect sufficient number of cells for transcriptional and methylation analysis, which also validated the global hypomethylation signature of these cells. Since the initiation of cell death and overall toxicity can affect gene expression and cell state, we do not attempt to infer the global effect of DNMT1 loss on hESCs. Instead, our results are only focused on the direct influences of DNMT1 perturbation on the methylation and activation of PEGs (which are normally hypermethylated and silenced in hpESCs), as they are not expected to be affected by DNA damage or cytotoxicity. Our results showed that PEGs can have diverse responses to global hypomethylation, calling for further investigation on the potential causes and mechanisms for such differences.

The CRISPR/Cas9 screen aimed at identifying loss-of-imprinting of the imprinted gene *PEG10*, revealed ATF7IP and ZMYM2 as regulators of *PEG10* and additional imprinted regions. ATF7IP was shown to bind to SETDB1 and to be essential for SETDB1-dependant H3K9me3 and gene silencing^44,45^. Proteomic analyses have revealed that ATF7IP directly interacts with the repressive HUSH complex, and that it can bind different partners by distinct protein domains^44,50,54^. Interestingly, a recent study has shown that ZMYM2 interacts with the C-terminal fibronectin type-III domain of ATF7IP and that it has a role in transgene silencing by ATF7IP^50^. The fact that our screen also identified both proteins independently, can suggest that they might also cooperate in silencing the maternal allele of *PEG10*. Nevertheless, exactly how ATF7IP and/or SETDB1 are specifically targeted to their associated genomic loci (including specific imprinted PEGs), remains mostly unknown and is an important open question. In addition to identifying specific regulators, the results of the screen also suggested involvement of the Mek/Erk pathway in regulating imprinting. Inhibiting this pathway (using high concentrations of PD0325901) confirmed upregulation of several PEGs in hpESCs. This is in accordance with previous studies in mEScs, which linked 2i culture conditions and specifically Mek/Erk inhibition or knockout with erosion of DNA methylation, including at imprinted control regions^41^.

Our results demonstrate a direct role for ATF7IP in preserving imprinting in humans. Studies in mice have previously associated histone methylation with imprinting regulation^55,56^, also specifying the involvement of *Setdb1* in this process^57^. Knockdown of *SETDB1* was shown to activate the PWS gene cluster on chromosome 15^58^, however our results indicated that there was only a small activation of this locus following *ATF7IP*-KO in hpESCs (Fig. 3c). Other studies pointed at the H3K9-methyltransferase G9a to be regulating the PWS locus^59,60^. Nevertheless, G9a was also reported to directly methylate lysine residues within ATF7IP, thereby affecting its repressive functions^54^. These conflicting reports prompt further investigations to pinpoint the roles of SETDB1/ATF7IP and G9a/GLP complexes in regulating repression of PEGs within the PWS locus, and to examine possible redundancy between them. ATF7IP and SETDB1 primarily act to repress endogenous retroviruses (ERVs)^61–63^, mainly during the global DNA demethylation post fertilization^62^. Insertion of ERVs was suggested to drive the evolution of parental imprinting^64–66^ and both *PEG10* and *FAM50B*, which were highly activated in *ATF7IP*-KO hpESCs, are derived from ancestral retrotranspositions^67–69^. Moreover, *FAM50B* is only imprinted in humans, whereas in mice it is biallelically expressed^70^. Consequently, our results propose that H3K9me3 set by ATF7IP and SETDB1, has evolved to regulate specific imprinted genes which are associated with retrotransposons, as part of its broader function to silence ERVs.

In *Drosophila*, SETDB1 and WDE (the homolog of ATF7IP) secure the identity of oocytes by repressing testis-specific genes^71^. In mice, SETDB1 is essential for meiosis in oocytes^72,73^ and for survival of spermatogonial stem cells^74^. Together with our findings that *ATF7IP* KO upregulates many sperm-specific genes in hESCs, SETDB1 and ATF7IP seem to have an evolutionary conserved role in regulating germ cell genes. Interestingly, aberrations in imprinted genes have been previously associated with sperm abnormalities^75^. Particularly, *FAM50B*, which we found to be regulated by ATF7IP, is highly expressed in normal spermatogenic cells^69^, whereas it features decreased methylation in asthenozoospermia^75^ and exhibit loss-of-imprinting in many seminomatous testicular germ cell tumors^70^. These indications prompt for further investigation regarding the involvement of ATF7IP in male infertility and testicular cancer via regulation of imprinted and non-imprinted genes that are important for spermatogenesis.

Collectively, our study uncovers a coupled role for ATF7IP in silencing sperm-specific genes, as well as a subset of maternally imprinted genes. Interestingly, both groups of genes are differentially regulated between oocyte and sperm, resulting in sex-specific germ cell development and formation of parental imprinting (Fig. 4d). Oocyte and sperm are known to possess eminent epigenetic asymmetries, which later persist in the maternal and paternal pronuclei following fertilization. Notably, spermatozoa chromatin is largely devoid of histones, thereby H3K9 methylation is absent throughout most of the paternal pronucleus^76^, allowing its rapid demethylation following fertilization^77^. In hESCs, ATF7IP maintains silencing of PEGs on the maternal allele, yet sperm-specific genes are repressed on both alleles (Fig. 4d). These differences call for further research to understand how the paternal allele is protected from ATF7IP-mediated repression, specifically at maternally imprinted genes. Finally, knocking out *ATF7IP* in hESCs results in epigenetic erasure of several maternally imprinted genes along with upregulation of sperm-specific genes (Fig. 4d).

## Methods

### Cell culture

Throughout the study we used the following cell lines: haploid hpESCs - hPES10^26^; diploid hpESCs - SwapS4^78^ (for RNA-Seq and RRBS), pES6, pES2, pES7^79^ (for DNA methylation array); bi-parental hESCs - WA09 (H9), CSES4 (for RNA-Seq), NYSCF1, NYSCF2, HuES53, HuES64 (for DNA methylation array).

hESCs were cultured on mouse embryonic fibroblast (MEF) treated with mitomycin-C. hESC growth medium was changed every 1–2 days, containing KnockOut Dulbecco’s modified Eagle’s medium (Gibco-Invitrogen, CA) supplemented with 15% KnockOut Serum Replacement (Gibco-Invitrogen, CA), 1 mM glutamine, 0.1 mM ß-mercaptoethanol (Sigma-Aldrich, MO), 1% nonessential amino acids stock (Gibco-Invitrogen, CA), penicillin (50 U/ml), streptomycin (50 μg/ml), and 8 ng/ml FGF2 (Gibco-Invitrogen, CA). Then, 10 μM ROCK inhibitor (Y27632, Stemgent) was supplemented to the medium in the first 24 h after thawing and passaging cells. Cells were passaged using short treatment with Trypsin-EDTA (Biological Industries, Beit Haemek, Israel).

### CRISPR/Cas9 library culture

Our recently established mutant library on haploid hpESCs (based on hpES10 cell line)^28^ was maintained for 4 weeks and frozen. Briefly, haploid-enriched cultures of hpES10 cell line were infected with a lentivirus library containing 181,131 sgRNAs at a multiplicity of infection of 0.3. Infected cells were selected with puromycin (Sigma) for 7 days. The mutant population was then expanded for about two weeks before it was frozen. The library was thawed and cultured at 37 °C with 5% CO_2_ in feeder-free conditions using Matrigel-coated plates (Corning) and mTeSR1 medium (STEMCELL Technologies) supplemented with 10 μM ROCK inhibitor (Y27632, Stemgent) for 1 day after thawing or splitting. Before reaching confluency, cells were passaged using Trypsin-EDTA (Biological Industries).

### Immunostaining and FACS sorting

To distinguish PEG10-expressing cells (PEG10^+^), we performed intracellular antibody staining. Cells were washed with phosphate buffered saline (PBS) and dissociated to single cells using Trypsin-EDTA. Following centrifugation, cells were resuspended in PBS and fixed by slow dropwise addition of MeOH until reaching 90% MeOH solution. The tubes were incubated for 30 min on ice and then cells were washed twice with PBS supplemented with 0.5% bovine serum albumin (BSA) and stained with anti-PEG10 antibody (1:500, Abcam ab215035) in 100% heat-inactivated fetal bovine serum (FBS) overnight at 4 °C. Cells were washed twice with PBS/BSA and stained with goat anti-Rabbit Alexa594-conjugated secondary antibody (1:800, Abcam ab150080) in 100% FBS for one hour on ice and washed twice again with PBS/BSA. Finally, the cells were filtered through a 70 μm cell strainer (Corning) and sorted using BD FACSAria III. We performed two separate immunostaining experiments, and for each experiment the cells were divided to two separate tubes which were sorted independently. Thus, we have considered the four different sorts as biological replicates. Each replicate started with ~150 million cells (>800 folds of the library), eventually sorting between 400,000 and 1,000,000 cells of PEG10-positive and PEG10-negative populations (~0.5% of the starting number of cells). In addition, ~30 million cells were harvested without sorting to serve as unsorted control.

### DNA sequencing and sgRNA enrichment analysis

Genomic DNA was extracted using gSYNC DNA extraction kit (Geneaid). A region containing the sgRNA integration was amplified with primers containing overhang sequences compatible for Nextera DNA library preparations (Illumina), as detailed in Supplementary Table 3 and as previously described^80^. After purification of the 160-base-pair (bp) product, a second PCR reaction was performed using Nextera adapter primers to generate a Nextera DNA library according to the manufacturer’s instructions (Illumina). DNA libraries containing sgRNA constructs from two replicate experiments were sequenced using NextSeq 500 (Illumina). For the unsorted controls, between 20 and 40 million cells were analyzed, corresponding to a coverage of ~100–200 fold of the library size. For the PEG10-positive and PEG10-negative conditions, between 400,000 and 1,000,000 were sorted and analyzed. The numbers of reads obtained from sequencing were 10–20 million for the unsorted controls, 3–7 million for the PEG10-positive and 2–10 million for the PEG10-negative cells. More than 150,000 sgRNAs were represented in the reads for unsorted controls, while the reads for the PEG10-positive and PEG10-negative conditions had a representation of 60,000–120,000 sgRNAs. edgeR^81^ was used to calculate the log_2_ fold change (Log_2_ FC) of sgRNA counts between unsorted control (*n* = 3 replicates) and PEG10-positive (*n* = 4 replicates) or PEG10-negative cells (*n* = 5 replicates). The differential representation analysis by edgeR was performed separately for every immunostaining experiment (each containing two replicates). Therefore, every gene incorporated FC values of ~20 sgRNAs (~10 sgRNAs from the library X 2 experiments). The final Log_2_ FC for a gene was calculated as the median of these sgRNAs after removing outliers (using the R command boxplot.stats(x)$out). As expected from a positive selection, most genes had a negative Log_2_ FC value. To normalize this bias, we centered the values around 0 by subtracting the median Log_2_ FC value of all the genes from the Log_2_ FC of each gene. The *P* value was calculated by two-sample, two-sided Kolmogorov-Smirnov test. Genes that passed the following filters were considered enriched and included in the “candidate gene” list (Supplementary Data File 1): (1) Log_2_ FC > 1.4 (equivalent to >0.5 before normalization); (2) *P* value < 0.05; (3) TPM > 1 (calculated from RNA-Seq performed previously on the CRISPR/Cas9 haploid hpESC library^30^); and (4) Genes having Log_2_ FC > 0.5 and *P* value < 0.05 in the PEG10-negative sort were discarded.

### RNA extraction and sequencing

Total RNA was isolated using RNeasy Mini Kit (QIAGEN) and mRNA was enriched by pull down of poly(A)-RNA. RNA sequencing libraries were generated using KAPA RNA Library Prep Kit according to the manufacturer’s protocol and sequenced using Illumina NextSeq 500 with 75 bp single-end reads. For analyses of gene expression, reads were mapped to the GRCh38 reference genome using STAR^82^. Normalization of the read counts, differential expression (DE) and statistical analyses were performed using edgeR^81^. The result of the RNA-Seq DE analysis can be found in Supplementary Data File 3.

### 5-azadC treatment

pES6 hpESCs were plated in a density of ~100,000 cells per well in a six-well plate and cultured for 24 h. 5-azadC (Sigma-Aldrich) was administered for 5 days in a final concentration of 2 or 5 μM. Cell media was exchanged every day, supplemented with fresh 5-azadC. Genomic-DNA was extracted and analyzed by Infinium 450K Methylation beadChips (Illumina) and RNA was extracted and analyzed by RNA sequencing.

### MEK/ERK inhibition

To block FGF2 signaling, SwapS4 hpESCs were cultured in standard hESC growth medium until reaching ~40% confluency. Then bFGF (FGF2) was removed from the medium with addition of 25 μM PD0325901 (Biogems #3911091) or 0.1% DMSO as control for 5 days. Medium was changed daily.

### Generation of individual gene knockouts

CRISPR/Cas9 with a specific sgRNA was used to target DNMT1 and five candidate genes. Sequences of these sgRNAs are listed in Supplementary Table 3. sgRNAs were cloned into the lentiCRISPR v2 lentiviral vector (a gift from Feng Zhang, Addgene cat. no. 52961). For control, we used lentiCRISPR v2 vector without any sgRNA. For viral production, 293T cells cultured in 10 cm culture plates with around 70–80% confluency were transfected with sgRNA-containing lentiCRISPR v2 (5.7 µg), pCMV-VSV-G (2.8 µg) and psPAX2 (4.3 µg) plasmids, in the presence of polyethylenimine “Max” (Polysciences). Transfection medium was exchanged with hESC medium after 24 h, and lentiviral particle-containing culture supernatant was harvested 60–65 h after transfection. Culture supernatant was spun down at 3000 r.p.m. for 10 min at 4 °C and then filtered through 0.45 µm cellulose acetate filters (Millipore). hPES10 or SwapS4 cells were trypsinized with Trypsin-EDTA, centrifuged, and resuspended in hESC growth medium supplemented with 10 μM ROCK inhibitor (Y27632) and 8 μg/ml polybrene (Sigma) and plated on feeder layer MEFs. Then, ~2 ml from the lentivirus-containing medium were then added to the six-well plates. At 24 h after transduction, virus-containing medium was replaced with standard hESC growth medium. At 36–48 h after transduction, the medium was replaced with medium that contains puromycin (0.3 μg/ml, Sigma). Cells were kept under antibiotic selection for 7–14 days, followed by extraction of RNA or DNA.

### ChIP-Seq analysis

For the analysis of H3K9me3, we reanalyzed previously published ChIP-Seq data performed in WT, *SETB1* KO, and *ATF7IP* KO HeLa cells^44^. Fastq files were downloaded to Galaxy^83^ via ftp link provided by European Nucleotide Archive (https://www.ebi.ac.uk/ena/browser/view/PRJNA342602) and aligned to hg38 using Bowtie2. BAM files were then converted to bigwig by bamCoverage (version 3.3.2) with bin size of 50 bp and visualized in the Integrated Genomics Viewer (IGV).

For the analysis of H3Ac, we used our recently published ChIP-Seq data performed in control and *ZMYM2*^−/−^ hESCs^49^.

### ChIP qPCR

ChIP assay was performed as described previously^84,85^ with slight modifications: In short, cells were crosslinked with 1% formaldehyde at RT for 10 min, quenched with 0.125 M glycine and scraped from plate. Samples were lysed, homogenized, and sonicated for 20 cycles (cycle = 30 s on and 30 s off). For immunoprecipitation, the samples were incubated with an antibody against H3K9me3 (Sigma-Aldrich, Cat#: 07-442) overnight and mixed with protein A beads the next day. Following washes, samples were eluted with TE and reverse-crosslinked at 65 °C. DNA was recovered with DNA cleanup kit (Qiagen) and 1 μl was used for qPCR with primers listed in Supplementary Table 3. For each gene (PEG10 and FAM50B), FCs of H3K9me3 levels in the DMR vs. gene body regions were calculated based on the qPCR results and normalized to control cells.

### DNA methylation analysis by methylation array

DNA methylation analysis of bi-parental and parthenogenetic hESCs treated with either DMSO (control) or 5-azadC for 5 days, was performed using Infinium 450K Methylation beadChips (Illumina) following the Infinium HD methylation protocol. Data was processed and normalized by using subset-quantile within array normalization (SWAN) and adjusted for batch effects using the R package ChAMP (version 1.4.0), as previously described^86^. Imprinted DMRs which were analyzed in this study and their association with CpG probes are listed in Supplementary Data File 4.

### DNA methylation analysis by reduced representation bisulfite sequencing

Genomic DNA was extracted from control, *ATF7IP* or *ZMYM2* KO hpESCs using gSYNC DNA extraction kit (Geneaid). From each sample, 1 µg of genomic DNA was sent to *CD Genomics* (Shirley, NY) for library preparation and sequencing. The DNA was digested with MspI (NEB), followed by ends preparation, adaptor ligation using Premium RRBS kit (Diagenode). Size selection was performed using AMPure XP beads (Beckman Coulter, Inc.) to obtain DNA fractions of MspI-digested products enriching for the most CpG-rich regions in the range of 150–350 bp. Subsequently, bisulfite treatment was conducted using the ZYMO EZ DNA Methylation-Gold Kit. The converted DNAs are then amplified by 12 cycles of PCR, using 25 μl KAPA HiFi HotStart Uracil+ ReadyMix (2X) and 8 bp index primers with a final concentration of 1 μM each and clean-up using AMPure XP beads. The constructed RRBS libraries were quantified by a Qubit fluorometer with Quant-iT dsDNA HS Assay Kit (Invitrogen), and sequenced on Illumina Hiseq platform using paired-end 150 bp strategy. Fastq files were uploaded to Galaxy^83^ and trimmed with Trim Galore! (http://www.bioinformatics.babraham.ac.uk/projects/trim_galore/) flagging the options --illumina and --RRBS. The trimmed Fastq files were aligned to GRCh38 using bwameth (https://github.com/brentp/bwa-meth) and methylation metrics were extracted using MethylDackel (https://github.com/dpryan79/MethylDackel), flagging the options --mergeContext, --counts, --logit, and --methylKit. The genomic coordinates of known imprinted DMRs^87^ were converted to GRCh38 using the LiftOver tool from UCSC (Supplementary Data File 4) and the methylation levels of CpGs within these regions were extracted using the BEDOPS bedextract command^88^. Finally, the mean methylation level for all CpGs of a given DMR was calculated. We filtered out maternal DMRs having mean methylation <0.6 or paternal DMR having mean methylation >0.4 in the control hpESCs.

### Analysis of tissue expression for genes upregulated in ATF7IP KO

The Expression Atlas website (https://www.ebi.ac.uk/gxa/home) was used to interrogate protein-coding genes that were significantly upregulated in *ATF7IP*-KO hpESCs (having log FC >2 and *P* value < 0.05), by downloading their TPM values across tissues taken from The Genotype-Tissue Expression (GTEx) database (https://gtexportal.org/home/).

### Reporting summary

Further information on research design is available in the Nature Research Reporting Summary linked to this article.

## Supplementary information

Supplementary information.

Reporting summary.

Description of Additional Supplementary Files.

Supplementary Data 1.

Supplementary Data 2.

Supplementary Data 3.

Supplementary Data 4.

## Data Availability

The CRISPR/Cas9 library sequencing, RNA-Seq and RRBS data generated in this study have been deposited in the ArrayExpress database under accession codes: E-MTAB-11012, E-MTAB-11014, and E-MTAB-11015. Previously published H3K9me3 ChIP-Seq data that was analyzed in this study is available under the following accession code: GSE86811. Previously published H3Ac ChIP-Seq data that was analyzed in this study is available under the following accession code: E-MTAB-8170
