# Supplementary material for: Identifying regulators of parental imprinting by CRISPR/Cas9 screening in haploid human embryonic stem cells
Source: Nat Commun. 2021 Nov 18;12:6718. doi: 10.1038/s41467-021-26949-7 (PMC8602306; doi:10.1038/s41467-021-26949-7)
Supplement: Supplementary file 1 — Supplementary information. [file 41467_2021_26949_MOESM1_ESM.pdf]

Supplementary Figure 1 - Global DNA demethylation in parthenogenetic human ESCs (related to Fig. 1)

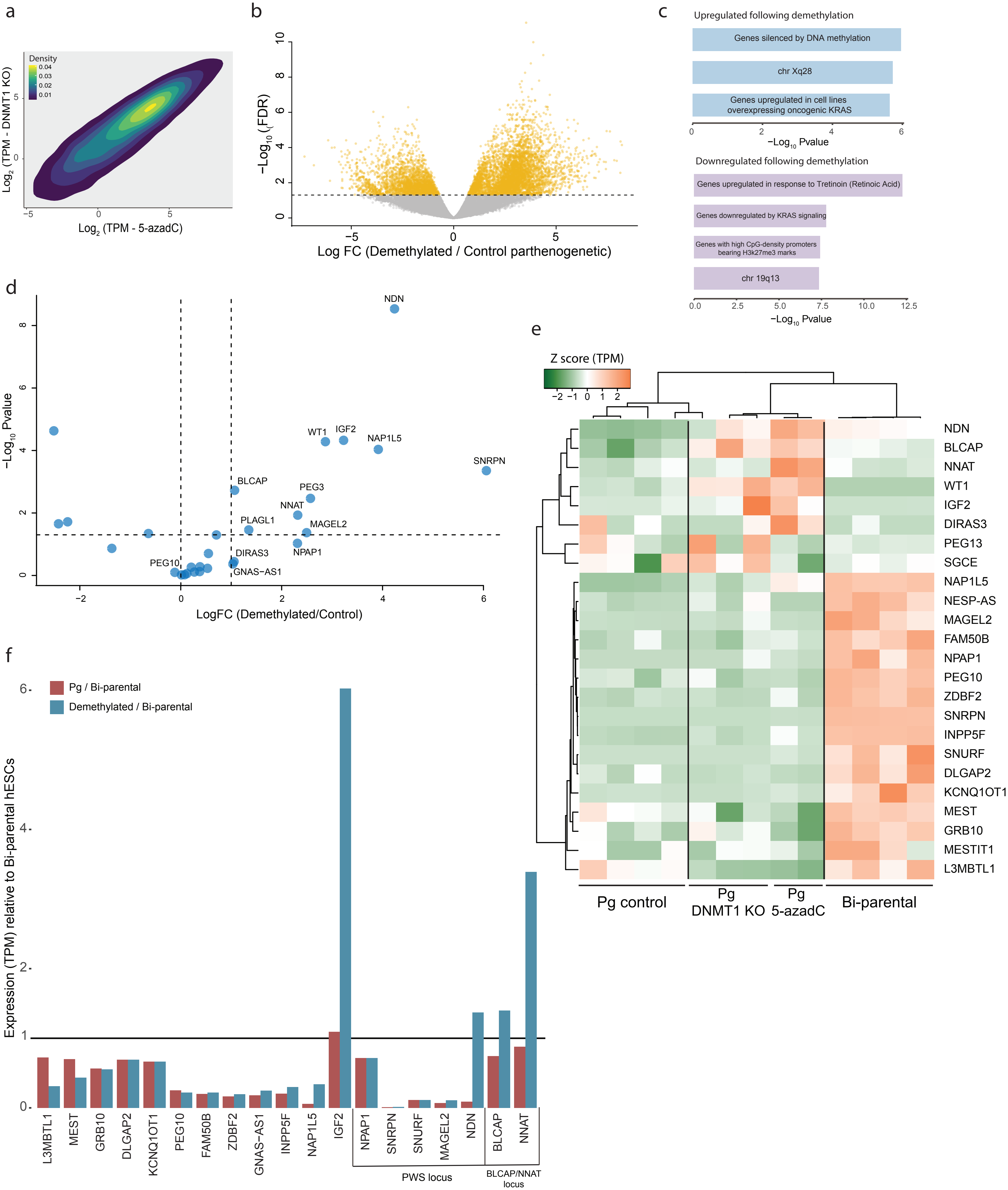

Supplementary Figure 1: Global DNA demethylation in parthenogenetic human ESCs (related to Fig. 1)

a, 2D density plot of log_2 TPM across all genes in *DNMT1* KO (y axis) vs. 5-azadC treated (x axis) parthenogenetic hESCs. **b,** Volcano plot of differential expression (calculated by edgeR) showing the log fold change (FC) between demethylated hpESCs (*DNMT1* KO samples (n = 3) and 5-azadC treated samples (n = 2)) and control (empty Cas9 vector samples (n = 3) and DMSO treated sample (n = 1)) for all genes (x axis). Y axis represents $-log_{10}$ FDR. **c,** Enriched GO terms (analyzed by GSEA, FDR q values < 0.05) of the upregulated (top, blue) or downregulated (bottom, purple) genes following demethylation (as identified in (b), logFC > 2 or logFC < -2 and FDR < 0.05). **d,** Same as (b), but illustrating only imprinted PEGs. **e,** Heatmap of expression levels (Z score TPM) of PEGs in bi-parental, control parthenogenetic, *DNMT1* KO and 5-azadC treated hpESCs. **f,** bar plot showing the fold change of the mean TPM in hpESCs for control samples (red, n = 5) or demethylated samples (blue, n = 4), relative to the mean TPM in bi-parental hESCs (n=4).

Supplementary Figure 2 - Loss-of-function screen in haploid hESCs reveals regulators of parental imprinting (related to Fig. 2)

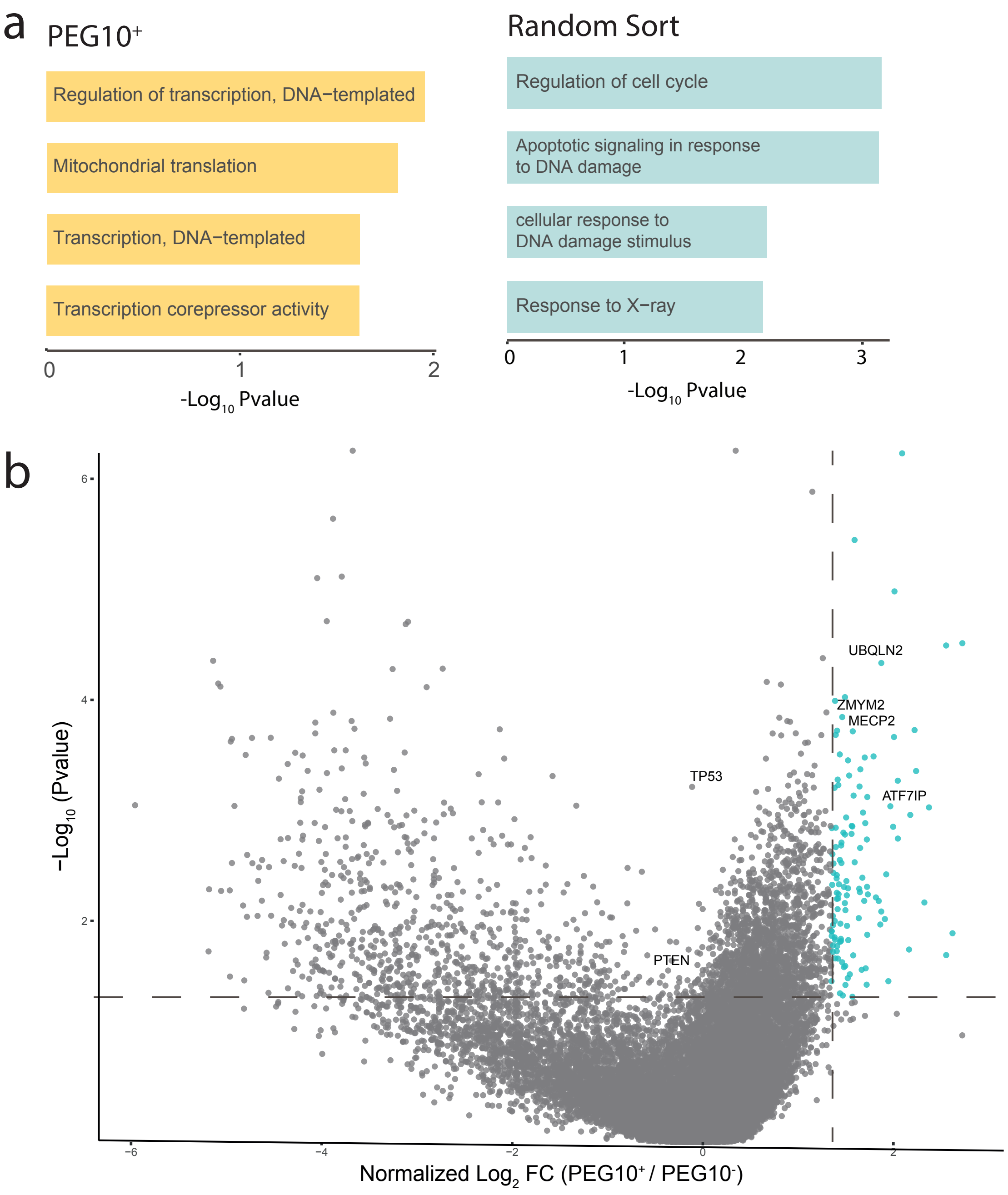

Supplementary Figure 2: Loss-of-function screen in haploid hESCs reveals regulators of parental imprinting (related to Fig. 2)

a, Enriched gene ontology (GO) terms (analyzed by DAVID) for significant genes with over-represented sgRNAs in $PEG10^+$ sorted cells (left, yellow) and PEG10-negative control (right, blue). **b,** Volcano plot showing the median $\log_2$ fold change (FC) of normalized sgRNA read counts (calculated by edgeR) per gene, between low coverage $PEG10^+$ and $PEG10^-$ populations (x axis, values are normalized to zero. n = 4 & 5 replicates). Y axis represents $-\log_{10}$ of the *P* value (two sample, two-sided Kolmogorov-Smirnov test). Marked in blue are enriched genes having normalized LogFC > 1.4 and *P* value < 0.05.

Supplementary Figure 3 - ATF7IP is required to maintain several maternally imprinted loci (related to Fig. 3)

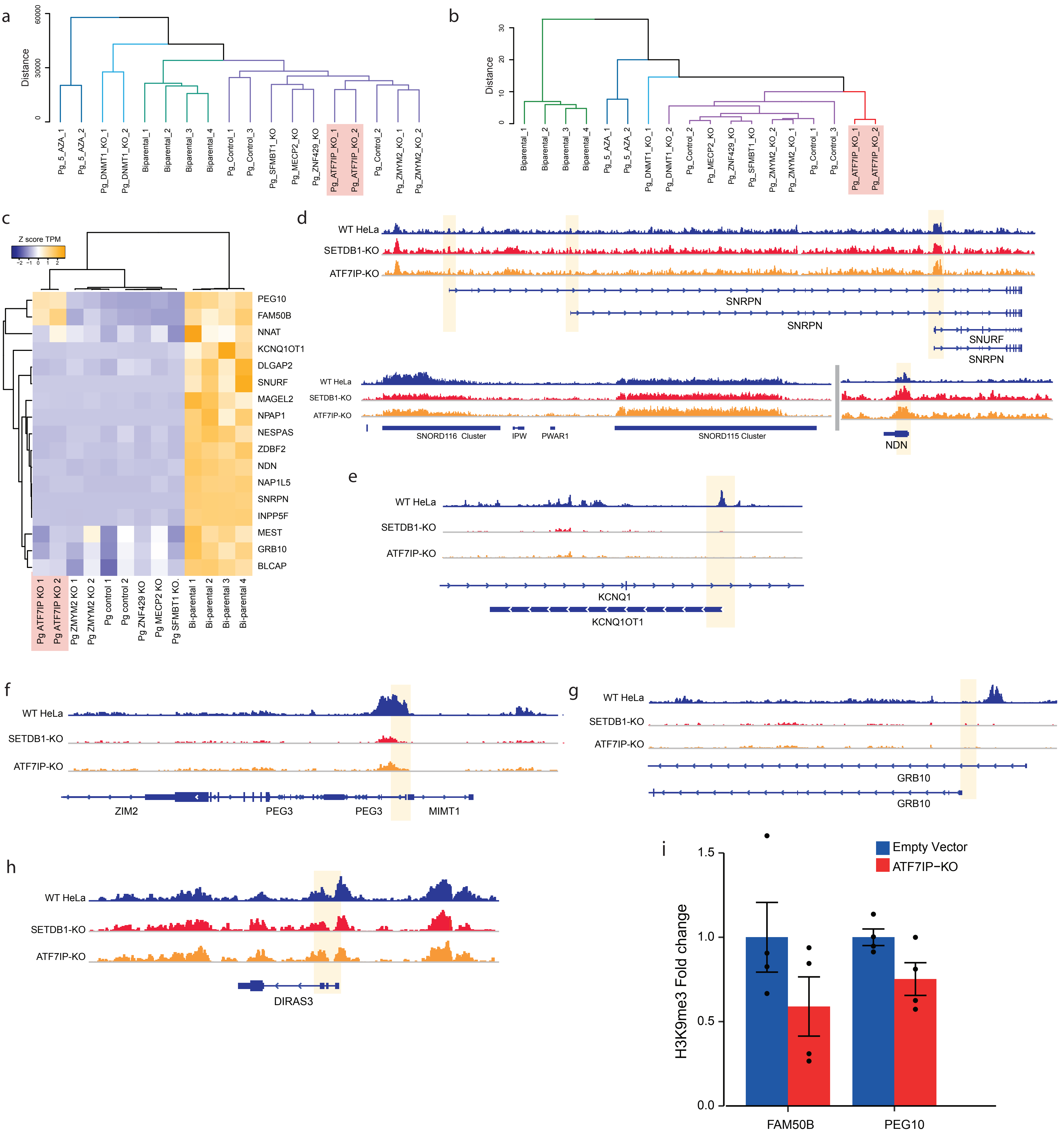

Supplementary Figure 3: ATF7IP is required to maintain several maternally imprinted loci (related to Fig. 3)

a, Hierarchical clustering ("Manhattan" distance measure) based on genome-wide expression levels (TPM) of bi-parental, control parthenogenetic, 5-azadC treated, DNMT1 KO and candidate gene KOs in hpESCs. **b,** Same as (a) but based only on expression of PEGs. **c,** Heatmap of expression levels (Z score TPM) of PEGs in bi-parental, control parthenogenetic and KOs of five candidate genes. **d-h,** Integrated genome viewer visualization of H3k9me3-ChIP-Seq peaks in WT, *SETDB1* KO and *ATF7IP* KO HeLa cells, at the PWS locus (d, three different regions are shown: *NDN*, *SNRPN*, *SNORD115-6*), *KCNQ1OT1* (e), *PEG3* (f), *GRB10* (g), *DIRAS3* (h). The location of the imprinted DMR is marked in yellow. **i**, Analysis of the effects of ATF7IP KO on H3K9me3 in *PEG10* and *FAM50B* DMRs in hpESCs. H3K9me3 ChIP qPCR showing the fold change compared to an adjacent control DNA region and relative to the empty vector. n=4 qPCR replicates. Data are presented as mean values +/- SEM.

Supplementary Figure 4 - ATF7IP KO induces DNA hypomethylation (related to Fig. 3)

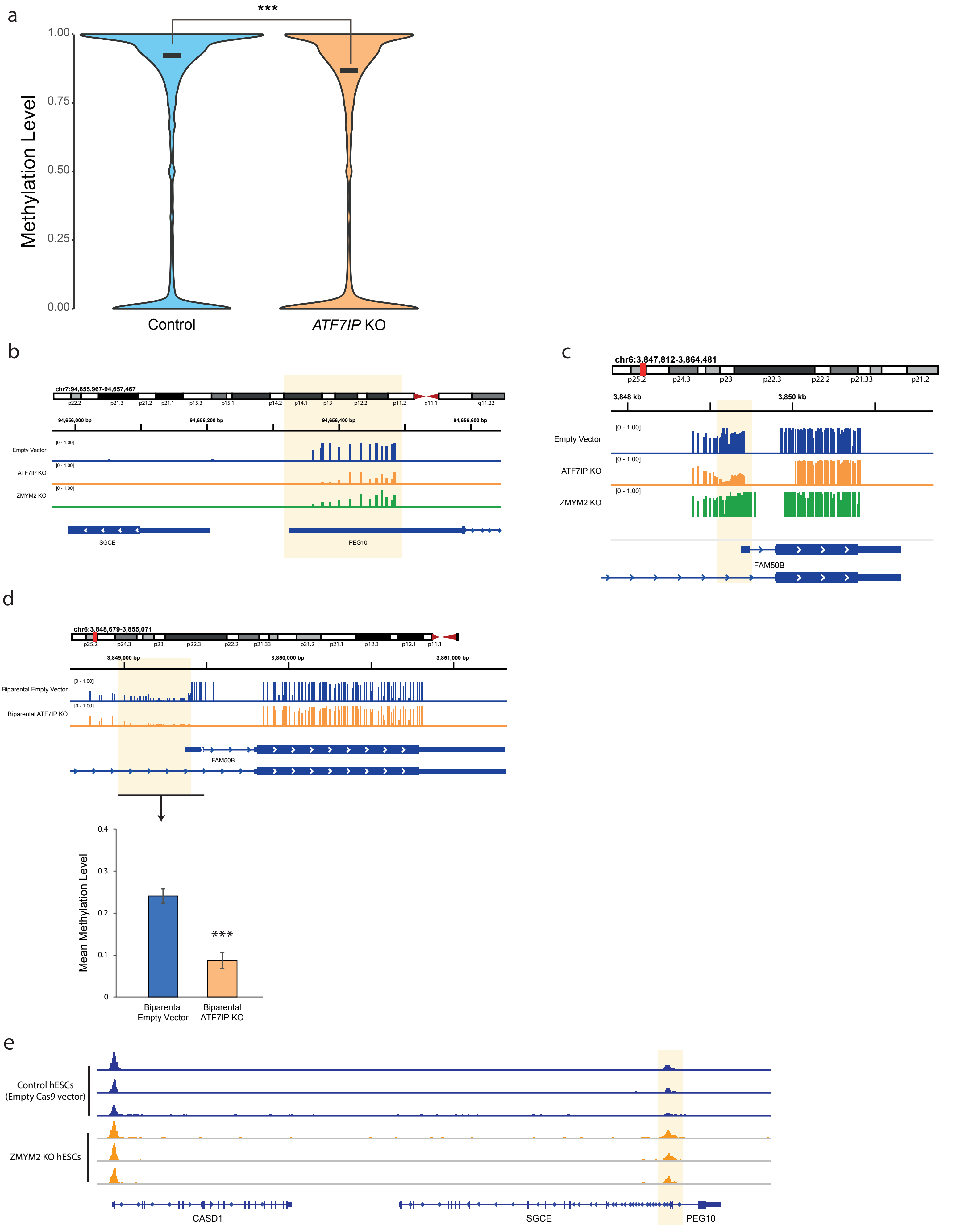

Supplementary Figure 4: ATF7IP KO induces DNA hypomethylation (related to Fig. 3)

a, Violin plot of global methylation levels (β values) across all CpGs analyzed by reduced representation bisulfite sequencing in control hpESCs (blue) and *ATF7IP* KO hpESCs (orange). ***$P < 2.2e\text{-}16$ (one tailed t-test). **b-c,** Integrated genome viewer (IGV) visualization of methylation values from RRBS in Control (empty vector), ZMYM2 KO and ATF7IP KO hpESCs at PEG10 (b) and FAM50B (c) loci. DMR region is highlighted in yellow. **d,** Top - Integrated genome viewer (IGV) visualization of methylation values from RRBS in Control (empty vector) and ATF7IP KO biparental hESCs at FAM50B locus. Bottom - a barplot indicating quantification of the mean methylation level across the DMR +/- SEM (P = 2.07e-7, one tailed t-test). **e,** Integrated genome viewer visualization of H3Ac-ChIP-Seq peaks in control (empty Cas9 vector) and *ZMYM2* KO biparental hESCs, surrounding the PEG10 locus.

Supplementary Figure 5 - ATF7IP KO induces expression of genes involved in spermatogenesis (related to Fig. 4)

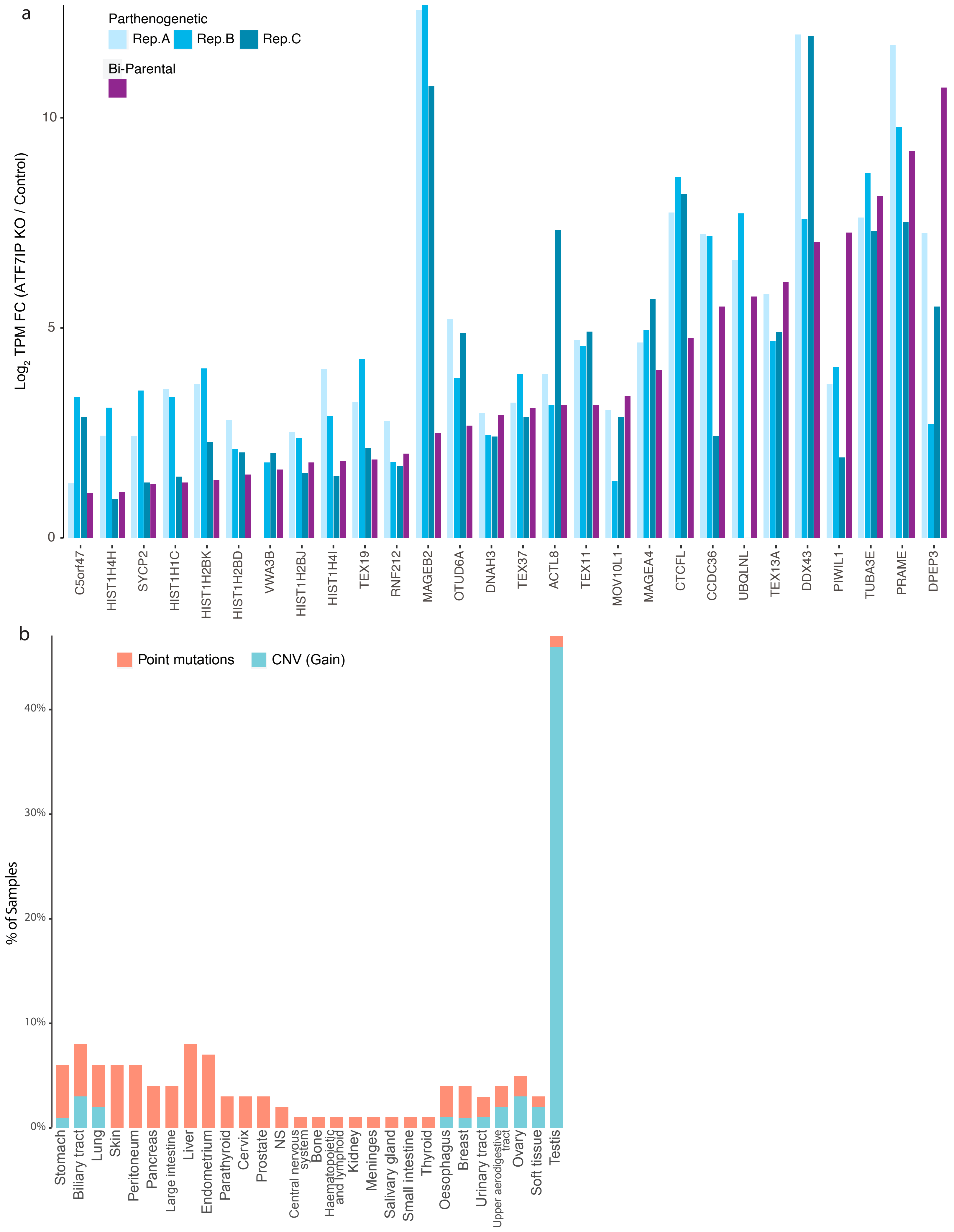

Supplementary Figure 5: ATF7IP KO induces expression of genes involved in spermatogenesis (related to Fig. 4)

a, Bar plot showing the log_2 expression fold change of representative sperm-specific genes between *ATF7IP* KO and control (empty Cas9 vector) in parthenogenetic (blue, n=3 independent experiments) and bi-parental (purple) hESCs. **b,** Bar plot showing the distribution of *ATF7IP* mutations across the primary tissue types as curated by the catalogue of somatic mutations in cancer (COSMIC) database. Red – point mutations, blue – copy number variation (CNV) gains.

Supllementary Table 1

Results of TFEA followed by KEA3 - Top 100

* - Genes in the top 20 results in both MeanRank and TopRank

Rank	Protein	Mean rank
1	MAPK1*	14.91
2	ATM	15.18
3	MAPK8	15.91
4	PRKDC*	18.55
5	HIPK2	18.91
6	CHEK2	20.27
7	CDK4*	22.18
8	CDK1	24.2
9	CDK7*	24.5
10	CSNK2A1*	25.18
11	CDK2*	27.73
12	GSK3B	28
13	ATR	28.4
14	CDK6*	29.3
15	MAPK9*	30.2
16	MAPK3*	31.73
17	CDK9*	34.73
18	AKT1	37.36
19	MAPK14*	40.45
20	PRKCD*	42.91
21	ABL1*	46.18
22	AURKA	48.36
23	NLK	52.3
24	PLK1	52.73
25	LCK	53.09
26	CSNK2A2	54.2
27	SYK	54.45
28	JAK2	55.55
29	IKBKB	55.73
30	MAPK7	56.3
31	CDK8	57.3
32	PIM1	57.9
33	SRC	59.64
34	VRK1	61.8
35	STK11	66.2
36	GSK3A	66.7
37	CHUK	66.82
38	MTOR	67.91
39	CDK5	68
40	CHEK1	69.64
41	TGFBR2	71
42	MAPKAPK2	75.44
43	LYN	76.09
44	IKBKE	79.09

45	MAPK11	79.45
46	MAP3K7	85.36
47	EGFR	86.36
48	CSNK1D	88.1
49	BTK	89.82
50	TGFBR1	92.56
51	PRKACA	92.64
52	RAF1	93.64
53	ERBB3	94.63
54	MAP2K1	94.64
55	HIPK1	95.22
56	TBK1	95.82
57	PRKCZ	97.09
58	JAK1	99.1
59	FGFR2	99.2
60	KIT	99.56
61	RPS6KA1	100.9
62	PLK3	101.5
63	PDGFRA	101.9
64	ERBB4	102.9
65	PRKCA	103.1
66	PDGFRB	106
67	MAP3K1	108.1
68	PRKCE	108.5
69	CSNK1A1	108.6
70	FGR	108.8
71	IGF1R	109.2
72	RPS6KA5	109.3
73	HIPK3	109.4
74	PAK1	109.9
75	PIK3CA	110.2
76	MAPK10	110.7
77	FYN	111.6
78	TTK	111.6
79	RNASEL	112
80	BUB1	112.4
81	BMX	112.7
82	IRAK1	112.8
83	DAPK3	112.9
84	ERBB2	113.4
85	PTK2	113.5
86	JAK3	114.3
87	EPHA3	114.8
88	AKT2	115.4
89	BUB1B	115.6
90	PRKAA1	116.6
91	PBK	117.7
92	HCK	120.7

93	RPS6KA3	120.8
94	TNK2	121.4
95	RET	121.6
96	NEK6	122.6
97	CSNK1E	125
98	PRKAA2	126.4
99	STK40	127.3
100	RPS6KB1	127.5

Supplementary Table 2

Diseases associated with ATF7IP

MalaCards: https://www.malacards.org/

MalaCards ID	Name	MalaCards information score	Score
GRM005	Germ Cell Cancer	47	3.766
TST026	Testicular Germ Cell Cancer	43	3.766
TST014	Testicular Cancer	46	3.702
CRY035	Cryptorchidism, Unilateral or Bilateral	58	2.618
CLR108	Colorectal Adenoma	64	0.101
ATM095	Autoimmune Disease	62	0.101
TST021	Testicular Germ Cell Tumor	60	0.101
LKM060	Leukemia, Acute Lymphoblastic 3	56	0.101
TRT010	Teratoma	52	0.101
GRM010	Germ Cells Tumors	34	0.101

Diseases: https://diseases.jensenlab.org/Search

	Name	Z-score	Confidence
Text mining	Cryptorchidism	4.4	★★★☆☆
	Testicular disease	4	★★★☆☆
	Primary hyperoxaluria type 1	3.8	★★☆☆☆
	Cancer	3.5	★★☆☆☆
	Hypospadias	3.2	★★☆☆☆
	Infertility	3.2	★★☆☆☆
	Male infertility	3	★★☆☆☆
	Gonadoblastoma	2.7	★★☆☆☆
	Seckel syndrome 7	2.5	★★☆☆☆
	Orofaciodigital syndrome IX	2.5	★★☆☆☆

	Name	Evidence	Confidence	Source
Experiments	Testicular cancer	p-value = 6e-10	★★★☆☆	DistiLD
	Breast cancer	p-value = 4e-08	★★★☆☆	DistiLD
	Carcinoma	54 samples	★★☆☆☆	COSMIC
	Lung cancer	14 samples	★☆☆☆☆	COSMIC
	Kidney cancer	12 samples	★☆☆☆☆	COSMIC
	Endometrial cancer	11 samples	★☆☆☆☆	COSMIC

DisGeNET: https://www.disgenet.org/home/

Disease_id	Disease	Score_gda	EI_gda	N_PMIDs	N_SNPs_gda	First_Ref	Last_Ref
C0039590	Testicular Neoplasms	0.4	1	2	1	2010	2013
C0751364	Cancer, Embryonal	0.3	1	1	0	2010	2010
C0027658	Neoplasms, Germ Cell and Embryonal	0.3	1	1	0	2010	2010
C0205851	Germ cell tumor	0.3	1	1	0	2010	2010
C0740345	Germ Cell Cancer	0.3	1	1	0	2010	2010
C0153594	Malignant neoplasm of testis	0.3	1	1	0	2010	2010
C1720811	Tumor of Rete Testis	0.3	1	1	0	2010	2010
C0023485	Precursor B-Cell Lymphoblastic Leukemia-Lymphoma	0.3	1	1	0	2014	2014
C0027654	Embryonal Neoplasm	0.3	1	1	0	2010	2010
C0205852	Neoplasms, Embryonal and Mixed	0.3	1	1	0	2010	2010
C0751365	Cancer, Embryonal and Mixed	0.3	1	1	0	2010	2010
C0036341	Schizophrenia	0.11	1	2	1	2014	2019
C1336708	Testicular Germ Cell Tumor	0.11	1	4	2	2013	2017
C0855197	Malignant Testicular Germ Cell Tumor	0.11	1	1	2	2010	2010

Supplementary Table 3<br>List of gRNAs and primers		
**Assay**	**Primer Name**	**Sequence**
Primers for Nextera DNA library	Nextera_Lib_F	TCGTCGGCAGCGTCAGATGTGTATAAGAGACAGNNNNNNNNNNGGCTTTATA- TATCTTGTGGAAAGGACG
	Nextera_Lib_R	GTCTCGTGGGCTCGGAGATGTGTATAAGAGACAGACGGACTAGCCTTATTT- TAACTTGC
CRISPR/Cas9 sgRNA	DNMT1 sgRNA	CACCGAGGCCAGAAGGAGGAACCG
	ATF7IP sgRNA	CACCGTGAAAAGGACAACAAACCTG
	ZMYM2 sgRNA	CACCGGGATATGATGAGTGAAGACG
	MECP2 sgRNA	CACCGGGAGCCTCTCACTGCAGTGA
	SFMBT1 sgRNA	CACCGGATGATGATTCCCTAAGTGA
	ZNF429 sgRNA	CACCGGAAAATTCATAGGATGGGTG
qPCR	PEG10_H3k9me3:	F: AGGACCTGGATTGGAACGAG<br>R: AGCTCCTCCTGAATGTGGTC
	PEG10_control:	F: AGTAGGGAGAGTGGGAGAGG<br>R: CCTCCTACCTCCCTACCCTT
	FAM50B_H3k9me3:	F: CATGCACCTCCTCAAGAAGC<br>R: GATGGTCTCCTCGGCGATG
	FAM50B_control:	F: CAGGTGATGCTTTGCCACTT<br>R: GGGCAGATAGGGAGAGAAGC
