# Supplementary material for: Identifying regulators of parental imprinting by CRISPR/Cas9 screening in haploid human embryonic stem cells
Source: Nat Commun. 2021 Nov 18;12:6718. doi: 10.1038/s41467-021-26949-7 (PMC8602306; doi:10.1038/s41467-021-26949-7)
Supplement: Supplementary file 2 — Reporting summary. [file 41467_2021_26949_MOESM2_ESM.pdf]

nature portfolio

Corresponding author(s): Nissim Benvenisty

Last updated by author(s): Oct 26, 2021

Reporting Summary

Nature Portfolio wishes to improve the reproducibility of the work that we publish. This form provides structure for consistency and transparency in reporting. For further information on Nature Portfolio policies, see our Editorial Policies and the Editorial Policy Checklist.

Statistics

For all statistical analyses, confirm that the following items are present in the figure legend, table legend, main text, or Methods section.

n/a	Confirmed	
☐	☒	The exact sample size (*n*) for each experimental group/condition, given as a discrete number and unit of measurement
☒	☐	A statement on whether measurements were taken from distinct samples or whether the same sample was measured repeatedly
☐	☒	The statistical test(s) used AND whether they are one- or two-sided<br>*Only common tests should be described solely by name; describe more complex techniques in the Methods section.*
☒	☐	A description of all covariates tested
☒	☐	A description of any assumptions or corrections, such as tests of normality and adjustment for multiple comparisons
☐	☒	A full description of the statistical parameters including central tendency (e.g. means) or other basic estimates (e.g. regression coefficient) AND variation (e.g. standard deviation) or associated estimates of uncertainty (e.g. confidence intervals)
☐	☒	For null hypothesis testing, the test statistic (e.g. *F*, *t*, *r*) with confidence intervals, effect sizes, degrees of freedom and *P* value noted<br>*Give P values as exact values whenever suitable.*
☒	☐	For Bayesian analysis, information on the choice of priors and Markov chain Monte Carlo settings
☒	☐	For hierarchical and complex designs, identification of the appropriate level for tests and full reporting of outcomes
☐	☒	Estimates of effect sizes (e.g. Cohen's *d*, Pearson's *r*), indicating how they were calculated

Our web collection on statistics for biologists contains articles on many of the points above.

Software and code

Policy information about availability of computer code

Data collection	*Provide a description of all commercial, open source and custom code used to collect the data in this study, specifying the version used OR state that no software was used.*
Data analysis	The following tools, softwares and R packages were used in this study: STAR (for RNA-Seq alignment), Bowtie2 (for ChIP-seq alignment), bamCoverage, EdgeR, ggplot2, dplyr, X2K Web, KEA3, IGV, Trim Galore!, bwameth, MethylDackel, UCSC LiftOver, BEDOPS bedextract, Expression Atlas website for extraction of GtEX data, DAVID, GSEA

For manuscripts utilizing custom algorithms or software that are central to the research but not yet described in published literature, software must be made available to editors and reviewers. We strongly encourage code deposition in a community repository (e.g. GitHub). See the Nature Portfolio guidelines for submitting code & software for further information.

Data

Policy information about availability of data

All manuscripts must include a data availability statement. This statement should provide the following information, where applicable:

- Accession codes, unique identifiers, or web links for publicly available datasets
- A description of any restrictions on data availability
- For clinical datasets or third party data, please ensure that the statement adheres to our policy

The CRISPR/Cas9 library sequencing, RNA-seq and RRBS data generated in this study have been deposited in the ArrayExpress database under accession codes: E-MTAB-11012, E-MTAB-11014 and E-MTAB-11015. Previously published ChIP-Seq data that was analyzed in this study is available under the following accession code: GSE86811.

Field-specific reporting

Please select the one below that is the best fit for your research. If you are not sure, read the appropriate sections before making your selection.

[x] Life sciences [ ] Behavioural & social sciences [ ] Ecological, evolutionary & environmental sciences

For a reference copy of the document with all sections, see nature.com/documents/nr-reporting-summary-flat.pdf

Life sciences study design

All studies must disclose on these points even when the disclosure is negative.

Sample size	For CRISPR screen, RNA-Seq experiments of KO or chemically treated cell lines, we used between 3-4 samples, which is considered a standard practice in the field.
Data exclusions	RNA-Seq samples that received low coverage (Less then 5M reads) were excluded from the analyses.
Replication	To assure reproducibility we KO the inspected genes in different cell types with different genetic backgrounds and made sure they replicated the same results. We aso included parthenogenetic and biparental cells as another layer of confidence.
Randomization	This was not relevant for our study, since no randomized experiment was performed.
Blinding	Blinding was not relevant for our study since no group allocation was performed.

Reporting for specific materials, systems and methods

We require information from authors about some types of materials, experimental systems and methods used in many studies. Here, indicate whether each material, system or method listed is relevant to your study. If you are not sure if a list item applies to your research, read the appropriate section before selecting a response.

Materials & experimental systems

n/a	Involved in the study	
[ ]	[x]	Antibodies
[ ]	[x]	Eukaryotic cell lines
[x]	[ ]	Palaeontology and archaeology
[x]	[ ]	Animals and other organisms
[x]	[ ]	Human research participants
[x]	[ ]	Clinical data
[x]	[ ]	Dual use research of concern

Methods

n/a	Involved in the study	
[x]	[ ]	ChIP-seq
[ ]	[x]	Flow cytometry
[x]	[ ]	MRI-based neuroimaging

Antibodies

Antibodies used	PEG10 antibody (Abcam, ab215035, 1:300 dilution)<br>Goat anti-Rabbit Alexa594-conjugated secondary antibody (Abcam, ab150080, 1:800 dilution)<br>H3K9me3 antibody (Sigma-Aldrich, Cat#: 07-442)
Validation	ab215035 from Abcam website - Knockout validated:<br>https://www.abcam.com/peg10edr-antibody-epr20051-ab215035.html?productWallTab=ShowAll<br>Cat#: 07-442 H3K9me3 antibody from Sigma Aldrich website - Use Anti-trimethyl-Histone H3 (Lys9) Antibody (rabbit polyclonal antibody) validated in DB, ICC, Mplex, PIA, WB, ChIP-seq to detect trimethyl-Histone H3 (Lys9) also known as H3K9me3, Histone H3 (tri methyl K9).:<br>https://www.sigmaaldrich.com/US/en/product/mm/07442

Eukaryotic cell lines

Policy information about cell lines

Cell line source(s)	We used haploid and diploid human embryonic stem cells which were previously reported and published.<br>Cell line names:<br>- H9 (Bi parental, XX) - commonly used human ES cell line.<br>- CSES4 (Cedars Sinai Embryonic Stem Cell 4, RRID:CVCL_B815), (Bi-parental, XX), reported in: https://doi.org/10.1634/stemcells.2008-0156<br>- PES10 (haploid, parthenogenetic), reported in: DOI: 10.1038/nature17408<br>- SWAPS4 (diploid, parthenogenetic), reported in: https://doi.org/10.1038/nature11800

	- PES6 (diploid, parthenogenetic), reported in: DOI:https://doi.org/10.1016/j.stem.2019.06.013
Authentication	Cell lines were validated for their ES and parental identity by gene expression. Haploid cells were validated by FACS.
Mycoplasma contamination	All cell lines tested negative for Mycoplasma
Commonly misidentified lines (See ICLAC register)	NA

Flow Cytometry

Plots

Confirm that:

- [x] The axis labels state the marker and fluorochrome used (e.g. CD4-FITC).
- [x] The axis scales are clearly visible. Include numbers along axes only for bottom left plot of group (a 'group' is an analysis of identical markers).
- [x] All plots are contour plots with outliers or pseudocolor plots.
- [x] A numerical value for number of cells or percentage (with statistics) is provided.

Methodology

Sample preparation	human ESCs were washed with phosphate buffered saline (PBS) and dissociated to single cells using Trypsin-EDTA. Following centrifugation, cells were resuspended in PBS and fixed by slow dropwise addition of MeOH until reaching 90% MeOH solution. The tubes were incubated for 30 minutes on ice and then cells were washed twice with PBS supplemented with 0.5% Bovine Serum Albumin (BSA) and stained with anti-PEG10 antibody (1:500, Abcam ab215035) in 100% heat-inactivated fetal bovine serum (FBS) overnight at 4°C. Cells were washed twice with PBS/BSA and stained with goat anti-Rabbit Alexa594-conjugated secondary antibody (1:800, Abcam ab150080) in 100% FBS for one hour on ice and washed twice again with PBS/BSA. Finally, the cells were filtered through a 70-µm cell strainer (Corning)
Instrument	BD FACSAria III
Software	FlowJo
Cell population abundance	NA
Gating strategy	Gating strategy was based on negative and positive controls, as shown in main figure 2

- [ ] Tick this box to confirm that a figure exemplifying the gating strategy is provided in the Supplementary Information.
