# Supplementary material for: Identifying regulators of parental imprinting by CRISPR/Cas9 screening in haploid human embryonic stem cells
Source: Nat Commun. 2021 Nov 18;12:6718. doi: 10.1038/s41467-021-26949-7 (PMC8602306; doi:10.1038/s41467-021-26949-7)
Supplement: Supplementary file 3 — Description of Additional Supplementary Files. [file 41467_2021_26949_MOESM3_ESM.pdf]

Description of Additional Supplementary Files

Supplementary Data 1:
List of genes identified in the CRISPR/Cas9 screen for PEG10 positive or negative sorts. Log_2 Fold change was calculated using EdgeR. *P* value was calculated by two sample, two-sided Kolmogorov-Smirnov test.

Supplementary Data 2:
Expression levels of genes that were upregulated in ATF7IP KO cells across multiple tissues from the genotype-tissue expression (GTEx) database.

Supplementary Data 3:
Sheets 1-2: Differential expression (DE) analysis between hpESCs carrying an empty Cas9 vector and DNMT1 or ATF7IP KO.
Sheet 3: TPM values for biparental cells carrying empty Cas9 vector or ATF7IP KO.

Supplementary Data 4:
Sheet 1: Genomic coordinates of imprinted DMR (hg38 genome build).
Sheet2: Details of Infinium 450K Methylation beadChips (Illumina) methylation probes which are found within imprinted DMRs.
